# PEGylated Paclitaxel Nanomedicine Meets 3D Confinement: Cytotoxicity and Cell Behaviors

**DOI:** 10.3390/jfb14060322

**Published:** 2023-06-15

**Authors:** Wenhai Lin, Yuanhao Xu, Xiao Hong, Stella W. Pang

**Affiliations:** 1 Department of Electrical Engineering, City University of Hong Kong, Hong Kong, China; 2 Centre for Biosystems, Neuroscience, and Nanotechnology, City University of Hong Kong, Hong Kong, China

**Keywords:** nanomedicine, microwell, paclitaxel, cell migration, 3D confinement

## Abstract

Investigating the effect of nanomedicines on cancer cell behavior in three-dimensional (3D) platforms is beneficial for evaluating and developing novel antitumor nanomedicines in vitro. While the cytotoxicity of nanomedicines on cancer cells has been widely studied on two-dimensional flat surfaces, there is little work using 3D confinement to assess their effects. This study aims to address this gap by applying PEGylated paclitaxel nanoparticles (PEG-PTX NPs) for the first time to treat nasopharyngeal carcinoma (NPC43) cells in 3D confinement consisting of microwells with different sizes and a glass cover. The cytotoxicity of the small molecule drug paclitaxel (PTX) and PEG-PTX NPs was studied in microwells with sizes of 50 × 50, 100 × 100, and 150 × 150 μm^2^ both with and without a concealed top cover. The impact of microwell confinement with varying sizes and concealment on the cytotoxicity of PTX and PEG-PTX NPs was analyzed by assessing NPC43 cell viability, migration speed, and cell morphology following treatment. Overall, microwell isolation was found to suppress drug cytotoxicity, and differences were observed in the time-dependent effects of PTX and PEG-PTX NPs on NPC43 cells in isolated and concealed microenvironments. These results not only demonstrate the effect of 3D confinement on nanomedicine cytotoxicity and cell behaviors but also provide a novel method to screen anticancer drugs and evaluate cell behaviors in vitro.

## 1. Introduction

Nanoparticles (NPs) have a wide range of applications, including catalysis, medicine, and sensors [1,2,3,4,5]. In medicine, nanomedicines can overcome the limitations of small-molecule drugs, such as strong hydrophobicity, poor bioavailability, and significant side effects [6,7,8,9]. Some nanomedicines, such as Abraxane and Genexol-PM, which are based on paclitaxel (PTX), are widely used in clinics to treat various cancers [10,11,12,13,14]. However, successful preclinical evaluation of nanomedicines requires 3D platforms, as 2D cell culture platforms on flat surfaces cannot mimic the complex 3D microenvironment in vivo.

To better test the cytotoxicity of small molecule drugs and nanomedicines in vitro, 3D platforms, such as microwells, have been used [15,16,17,18,19]. Microwells have been widely used in cancer cell studies, including immune cell-mediated cancer treatment and the evaluation of anticancer drug response [20,21,22]. They have also been utilized to help cancer cells form spheroids to test the cytotoxicity of nanomedicines [23,24]. However, most of these studies have primarily focused on the collective influence of nanomedicines on the cancer cell population. Previous research has uncovered a clear correlation between the susceptibility of cancer cells to treatments and microwell size [25,26]. Therefore, it is crucial to assess the impact of physical confinement by microwells on the effectiveness of nanomedicines at the single-cell level to better understand the time-dependent nanomedicine treatment on individual cancer cells. To the best of our knowledge, this is the first study to use 3D confinement with a cover on top to evaluate the cytotoxicity of nanomedicine and cell behaviors after cancer cells are treated with nanomedicines.

**Figure 1:**
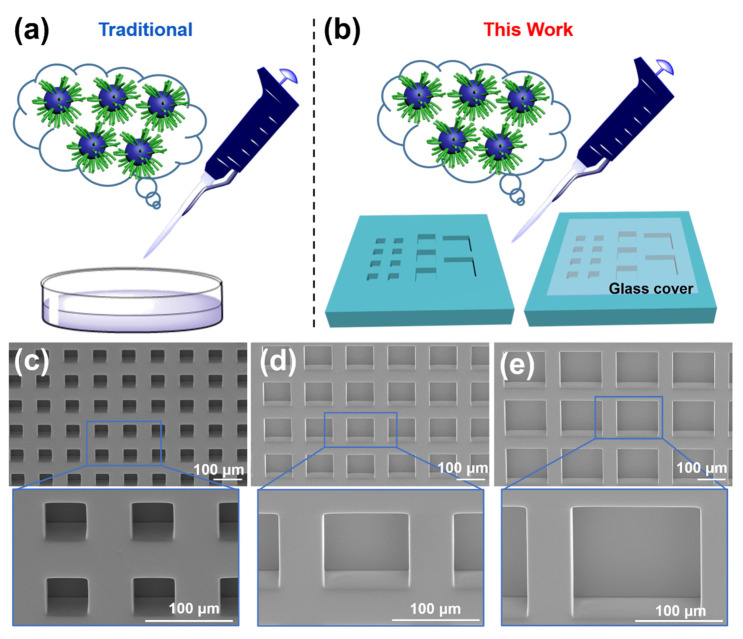
Schematic illustration to study effect of nanomedicines on cell behaviors and cytotoxicity of PEG-PTX NPs on (**a**) flat surface and (**b**) 3D platforms of microwell arrays without and with glass cover. Green: PEG; Blue: PTX. Scanning electron micrographs of (**c**) 50 × 50 μm^2^, (**d**) 100 × 100 μm^2^, and (**e**) 150 × 150 μm^2^ polydimethylsiloxane microwells.

Typically, a 2D flat surface, such as a petri dish, is used to study the effect of nanomedicines on cell behaviors, as shown in Figure 1a. However, in this study, 3D platforms consisting of microwells with different sizes and glass covers were used, as shown in Figure 1b. The covers provided additional concealment to the microwells from the top, mimicking the hindered drug delivery to the inner regions of a tumor. The microwells had flat bottoms instead of curved ones, enabling the evaluation of cell migratory behavior. PTX has been identified as a candidate for the treatment of nasopharyngeal carcinoma (NPC), but additional measures are needed to suppress its side effects and enhance its delivery efficiency [27,28]. For the first time, PEGylated paclitaxel nanoparticles (PEG-PTX NPs) were applied to treat NPC43 cells in microwells without and with a cover. In the early stages of NPC development, cancer cell clusters typically have diameters ranging from 50–250 µm [29,30]. To replicate this condition, we selected three different microwell sizes of 50 × 50 µm^2^, 100 × 100 µm^2^, and 150 × 150 µm^2^ to investigate how different levels of confinement affect the behavior of NPC43 cells when treated with drugs. The impact of 3D microwell isolation and glass cover concealment on the cytotoxicity of PTX and PEG-PTX NPs towards NPC43 cells was studied and compared with traditional 2D cell culture models. The varied time-dependent effects of the two drugs in confined 3D microenvironments on NPC43 cells were analyzed in terms of cell viability, cell migration speed, and cell morphology changes over specified time periods.

## 2. Materials and Methods

### 2.1. Materials

Polydimethylsiloxane (PDMS) pre-polymer (SYLGARD™ 184) was purchased from Dow (Midland, MI, USA); 97% Trichloro (1*H*, 1*H*, 2*H*, 2*H*-perfluorooctyl) silane (FOTS) was purchased from J&K Scientific (Beijing, China); 99.99% Tetrahydrofuran (THF) and 99.8% dimethylformamide (DMF) were purchased from Merck (Rahway, NJ, USA); 99.5% Paclitaxel was purchased from Xi’an Haoxuan Biological Technology Co., Ltd. (Xi’an, China). PEGylated (methoxypolyethylene glycol 2000) paclitaxel (PEG-PTX) was synthesized according to the previous literature [25,26,31]. Tubulin-tracker green and thiazolyl blue tetrazolium bromide (MTT) assays were purchased from Beyotime (Nantong, China).

### 2.2. Characterization

The diameter and size distribution of PEG-PTX NPs were measured using dynamic light scattering (Zeta-sizer Nano, Malvern, UK). The migration of cells, cell division, and cell disruption were recorded using an optical microscope (Eclipse, Nikon, Tokyo, Japan). The fluorescence of tubulin in cells was measured using a confocal laser scanning microscope (TCS SP5, Leica, Wetzlar, Germany). The morphologies of microwells, nanoparticles, and cells were measured using a scanning electron microscope (SU5000 FE, Hitachi, Tokyo, Japan). Cell viability was determined by measuring the absorbance at 490 nm using a microplate reader (Bio-Rad, Hercules, CA, USA). The migration speed, migration trajectories, cell area, and aspect ratio of cells were calculated using Image J software (version 1.53a). The aspect ratio of the cell shape was determined by fitting an ellipse over the cell, with a short axis a and a long axis b, to obtain the ratio b/a.

### 2.3. Fabrication of Microwell Arrays

PDMS microwell arrays were fabricated using previously reported methods [24]. Si was used to fabricate the mold of microwell arrays. Microwells were patterned on Si wafers using photolithography with AZ6130 as the photoresist. Then, 50 μm tall pillars in Si were etched using a deep reactive ion etching system. The etch conditions consisted of a repeated 7 s passivation cycle with 85 sccm C_4_F_8_, 600 W coil power at 20 mTorr, and a 14 s etch cycle with 130 sccm SF_6_, 600 W coil power, 20 W platen power at 40 mTorr. Thirty-four alternative cycles were applied to fabricate the 50 μm tall pillars. A monolayer of FOTS was coated on the Si mold as anti-stiction layer. The PDMS pre-polymer with a 10:1 base-to-curing agent ratio was cast on the Si mold, spin-coated at 1000 rpm for 1 min, and cured at 80 °C overnight. Replicated microwell arrays were then peeled off from the Si mold for further processing.

### 2.4. Synthesis of PEG-PTX NPs

A similar method was used as described in our previous work [32,33,34]. Briefly, PEG-PTX (40 mg) was dissolved in THF (5 mL) and dropwise added into water (10 mL) under stirring. After THF was completely evaporated, PEG-PTX NPs were collected.

### 2.5. Cell Culture

NPC43 cells were cultured in Roswell Park Memorial Institute 1 × 1640 medium (Gibco, Franklin Lakes, NJ, USA) with 10% fetal bovine serum (FBS, Gibco, USA), 0.2% 2 mM rock inhibitor Y-27632 (ENZO, Broomfield, CO, USA), and 1% antibiotic antimycotic (Gibco, USA). NPC43 cells were maintained in an incubator at 37 °C with 5% CO_2_. The medium was changed every two days, and the cells were passaged when they reached 70% confluency.

### 2.6. Cell Viability Assays

MTT assays against NPC43 cells were carried out using a similar experimental process as described in our previous work [35,36]. Briefly, PTX and PEG-PTX NPs were added to the cell culture medium at different concentrations. NPC43 cells were incubated with PTX and PEG-PTX NPs for 16, 40, and 64 h.

### 2.7. Time-Lapse Imaging

Microwell platforms with different sizes were bonded to a confocal dish using O_2_ plasma. After two sterilizations with 70% ethanol, the confocal dish with platforms was placed in a plasma system and treated for 5 min with O_2_ plasma to form a hydrophilic surface. The O_2_ plasma conditions were 20 sccm flow rate, 100 mTorr chamber pressure, and 100 W RF power. The dish was maintained in 1× phosphate buffered saline (PBS) before cell seeding. A total number of 1 × 10^5^ NPC43 cells were seeded into the dish for each experiment to have 1 cell in 50 × 50 μm^2^ microwells, 2–5 cells in 100 × 100 μm^2^ microwells, and 5–15 cells in 150 × 150 μm^2^ microwells. The cell-seeded microwell platforms were maintained in cell culture medium for 8 h incubation to form good cell adhesions. After that, the medium was changed to a mixture of 1:1 complete cell culture medium and CO_2_ independent medium (Gibco, USA) supplemented with 10% FBS, 1% antibiotic antimycotic, and 1% 100× GlutaMax (Gibco, USA). Then, PTX (5.85 nmol/mL) or PEG-PTX NPs (5.85 nmol/mL) was added. The PDMS chips were completely submerged in medium. In half of the samples, cells were seeded in PDMS microwells with an open top. Cancer cells were isolated into small colonies by the sidewalls, while additional nutrients and drugs could be supplied from the medium above the microwells as they were consumed over time. In the other samples, glass covers were placed on top of the PDMS chips to conceal the microwells. In these concealed microwells, the exchange of content between the inside of the microwells and the external environment was severely hindered. As a result, drug depletion in the concealed microwells was faster than in the open microwells, which more closely resembled the scenario for cancer cells in actual tumors with physical confinement and restricted drug delivery from the surrounding microenvironments.

A Nikon Eclipse upright microscope was used to capture cell movements for 16 h at 5 min intervals. To prevent fast cell culture medium evaporation, the imaging chamber was continuously supplied with humidified air.

### 2.8. Cell Preparation for Scanning Electron Microscopy

Cells on platforms were washed twice with PBS and fixed for 15 min with 4% paraformaldehyde. The cells were then treated with ethanol at concentrations of 30%, 50%, 70%, 80%, 90%, 95%, and 100% for 5 min each, and then dried in a critical point dryer (EM CPD300, Leica, Germany) for 4 h. To prevent charging during imaging in the scanning electron microscope (SEM, SU5000 FE-SEM, Hitachi, Japan), a thin layer of Au was coated onto the dried samples using a thin film coater (Q150 coater, Quorum Technologies, Lewes, UK). The coated samples were then mounted onto a 51 mm stub using conductive tape. Samples without and with cells were captured using a secondary electrons detector with a 10 kV accelerating voltage to achieve high-resolution images.

## 3. Results and Discussions

### 3.1. Characterization of Microwells and PEG-PTX NPs

Polydimethylsiloxane (PDMS) microwell arrays were fabricated following our previous work [31], with microwells of different sizes, as shown in Figure 1c–e. The microwells had dimensions of 50 × 50, 100 × 100, and 150 × 150 μm^2^, with a depth of 50 μm and a 50 μm space between adjacent microwells. These microwells were utilized as 3D platforms to assess the impact of PEG-PTX nanomedicine on NPC43 cells.

**Figure 2:**
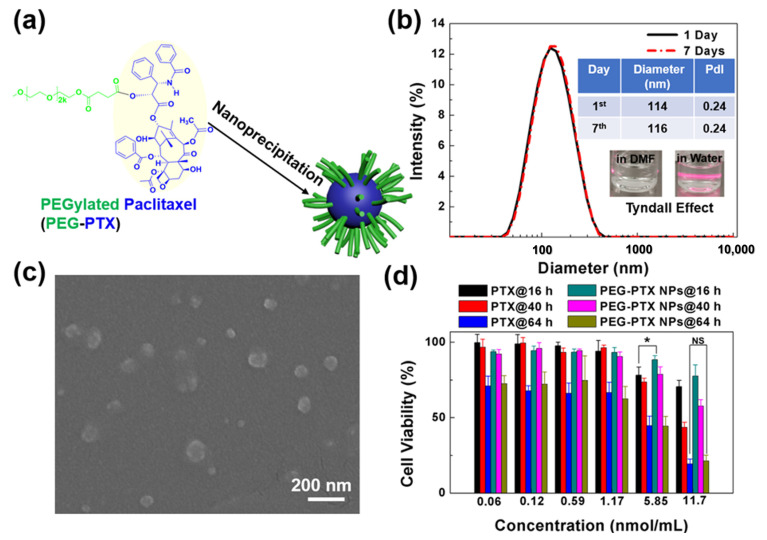
Characterization of PEGylated paclitaxel nanoparticles (PEG-PTX NPs). (**a**) Schematic illustration of PEG-PTX NPs synthesis. (**b**) Size distribution and 7-day stability of PEG-PTX NPs measured by dynamic light scattering. Inserted images: PEG-PTX dissolved in dimethylformamide (**left**) and PEG-PTX NPs in water (**right**). (**c**) Scanning electron micrographs of PEG-PTX NPs. (**d**) Cell viability of NPC43 cells treated with PTX and PEG-PTX NPs for 16, 40, and 64 h, respectively. One-way ANOVA and Tukey’s post-hoc tests, NS—not significant; * *p* < 0.05.

PEG-PTX was synthesized following previously reported methods [37,38,39]. Due to the hydrophilic nature of PEG and the hydrophobicity of PTX, the amphiphilic PEG-PTX could self-assemble into nanoparticles (PEG-PTX NPs) in water using the nanoprecipitation method, as shown in Figure 2a. Due to its nature as a prodrug, PEG-PTX does not experience drug loading efficiency issues when self-assembled into NPs. The content of PTX in PEG-PTX is 29% based on molecular weight, and this value remains consistent in NP form, as no additional compounds are introduced during the self-assembly process. As a result, PEG-PTX NPs offer a reliable and effective means of delivering PTX to cancer cells. The diameter and polydispersity index (PdI) of PEG-PTX NPs were measured using dynamic light scattering (DLS), with results shown in Figure 2b, indicating a diameter of 114 nm and a PdI of 0.24. After storage at room temperature for 7 days, the diameter and PdI of PEG-PTX NPs remained unchanged, indicating that the synthesized PEG-PTX NPs were stable. The stability of PEG-PTX NPs in cell culture medium (Roswell Park Memorial Institute 1 × 1640 medium with 10% fetal bovine serum, 0.2% 2 mM rock inhibitor, and 1% antibiotic antimycotic) for 24 h was measured by DLS, as shown in Supplementary Figure S1. The unchanged diameter and PdI showed favorable colloidal stability of PEG-PTX NPs in the complex cell culture medium. The inserted photos in Figure 2b demonstrate the clear Tyndall effect of PEG-PTX NPs in water, as a bright light path was observed when irradiated by a laser, which indicated successful nanoparticle synthesis in water. However, since PEG-PTX could dissolve in DMF, no nanoparticles or Tyndall effects were observed in DMF. The morphology of PEG-PTX NPs was revealed using scanning electron microscopy (SEM), as shown in Figure 2c, where some PEG-PTX NPs had a diameter below 100 nm. Overall, the PEG-PTX NPs had a slightly smaller size than that measured by DLS, as shown in Figure 2b, due to the use of a hydration layer in DLS but a dry sample in SEM. These results demonstrate successful formation of stable PEG-PTX NPs for biological experiments.

### 3.2. Cytotoxicity of PEG-PTX NPs in Microwells

#### 3.2.1. PEG-PTX NPs Followed Same Working Mechanism in Microwells as on Open Regions

The chemotherapy effect of PTX and PEG-PTX NPs on NPC43 cells was tested using thiazolyl blue tetrazolium bromide (MTT) assays, revealing time- and concentration-dependent cytotoxicity, as shown in Figure 2d. The concentration of NPs shown in this study was the equivalent molar concentration of PEG-PTX prodrug. Based on the molecular structure of PEG-PTX, the linker between PTX and PEG is an ester bond that is susceptible to destruction by esterase and the acidic microenvironment present in cancer cells. Therefore, after the NPs are endocytosed by cancer cells, they will be disassembled in the complex cancer microenvironment, and the ester bond in PEG-PTX will be destroyed, releasing PTX. This mechanism of drug release ensures that PTX is delivered directly to cancer cells and reduced the potential for undesirable side effects [37,38]. As time and concentration increased, cell viability decreased after treatment with PTX or PEG-PTX NPs, indicating the potential efficacy of PEG-PTX NPs in treating NPC43 cells. At a 16 h incubation time, small molecule PTX was more toxic than PEG-PTX NPs, as PTX had a direct drug effect, but PEG-PTX NPs required time to release PTX to cells. However, after 64 h of incubation, both PEG-PTX NPs and PTX displayed similar chemotherapy effects toward NPC43 cells, indicating the effectiveness of PEG-PTX NPs as an anticancer nanomedicine. The half-maximal inhibitory concentrations (IC_50_) of PTX and PEG-PTX NPs towards NPC43 cells for 64 h were 1.41 and 1.65 nmol/mL, respectively.

For its therapeutic effect, PTX can promote the assembly and polymerization of tubulin, which will result in the inhibition of mitosis and motility. Hence, to investigate the effects of PTX and PEG-PTX NPs on NPC43 cells, the microtubules of treated NPC43 cells were marked using tubulin-tracker green assays after being seeded in PDMS microwells and treated with PTX or PEG-PTX NPs for 16 h. To ensure consistency across subsequent experiments, a fixed concentration of 5.85 nmol/mL was used for both PTX and PEG-PTX NPs. This concentration was selected based on the results of MTT assays, which showed that the difference in cytotoxicity between PTX and PEG-PTX NPs was more pronounced within 16 h of treatment due to the timed release of NP drugs. By using a consistent concentration of PTX and PEG-PTX NPs in microwells of different sizes, the effect of these drugs on NPC43 cells in a confined environment can be compared. At a molar concentration of 5.85 nmol/mL, PEG-PTX would release the same amount of PTX as directly adding 5.85 nmol/mL of free PTX after the NPs were completely disassembled.

**Figure 3:**
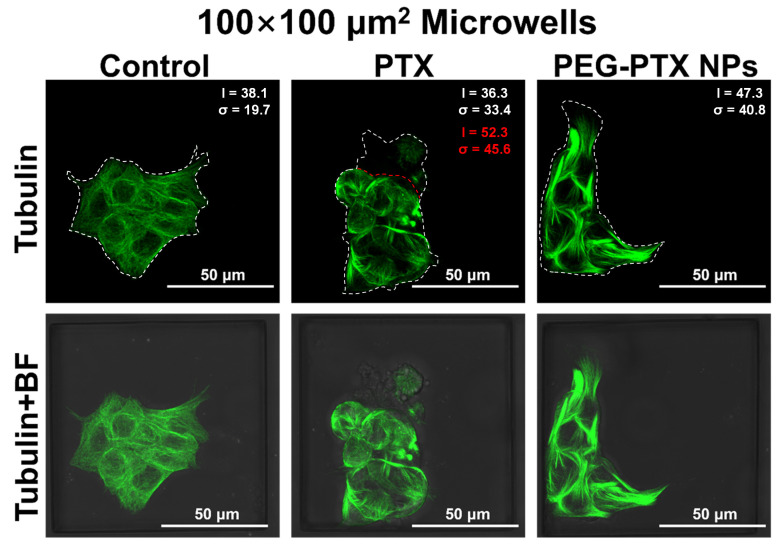
Confocal laser scanning micrographs of NPC43 cells incubated with PTX and PEG-PTX NPs for 16 h in 100 × 100 μm^2^ microwells. Tubulin in cells was stained by tubulin-tracker green (green fluorescence) and overlays of tubulin and bright field (BF) images. Average value (I) and standard deviation (σ) of fluorescent intensity were larger after drug treatment. Red dotted lines outlined the boundary between ruptured cells and intact cells. Statistics presented in red excluded the ruptured cells. Concentration of PTX and PEG-PTX NPs were both 5.85 nmol/mL.

As shown in Figure 3, NPC43 cells in 100 × 100 μm^2^ microwells without treatment, as a control group, exhibited uniform and network-like green fluorescence, indicating that microtubules in the control group were well-organized and reticular to maintain the stretched shape of cells. However, after treatment with PTX for 16 h, NPC43 cells became rounded, and microtubules were assembled together, resulting in bright and heterogeneous green fluorescence. For cells treated with PEG-PTX NPs for 16 h, bright green fluorescence of the tubulin bundles was observed, as microtubules were gathered into large clusters, but some cells still had an elongated shape. The average fluorescent intensity (I) and the standard deviation of fluorescent intensity (σ) within the NPC43 cells were calculated and compared. The control experiment, which involved no drug treatment, yielded I of 38.1 and σ of 19.7. In the presence of PEG-PTX NPs, NPC43 cells exhibited larger I and σ values of 47.3 and 40.8, respectively, likely due to bundled microtubules. In the case of PTX-loaded microwells, some NPC43 cells lost their fluorescent signal due to ruptured cell membranes. For the remaining cells, I and σ were 52.3 and 45.6, respectively, which were both significantly larger than those of the control group and slightly larger than the values observed for PEG-PTX NPs. Supplementary Figures S2 and S3 show microtubule images of NPC43 cells in 50 × 50 μm^2^ and 150 × 150 μm^2^ microwells, respectively, and reveal similar trends in fluorescent intensity statistics. These findings suggest that PTX released from PEG-PTX NPs is capable of acting on intracellular microtubules. Additionally, the results indicated that PTX is more toxic than PEG-PTX NPs for NPC43 cells during the first 16 h of treatment, as evidenced by a more rounded cell morphology, a higher proportion of ruptured cells, and a higher ratio of assembled microtubules with larger σ values. 

The overall fluorescent intensity was slightly lower in 50 × 50 μm^2^ microwells, which could be attributed to a lower cell staining efficiency resulting from the higher degree of microwell confinement. However, no significant morphological differences were observed in the microtubules of NPC43 cells across microwells of different sizes, suggesting that the NP intake and working mechanism were not affected by microwell confinement.

#### 3.2.2. Reduced Overall Cytotoxicity with Microwell Confinement and Isolation

To evaluate the effect of the microwell size and the degree of confinement on the cytotoxicity of PTX and PEG-PTX NPs, the percentage of cell division and cell disruption were calculated as they represented the cellular activity and cell death. In this study, cell division was identified as a single cell finishing its proliferation cycle and dividing into two individual cells. Cell disruption was identified by observing a cell that visibly exploded, became dimmer with an unsmooth surface, and became immobile. After seeding NPC43 cells in the microwells and incubating with PTX or PEG-PTX NPs, a glass cover was placed on the top of the microwells to provide additional confinement and limit the exchange of nutrients and drugs between the microwells and the surrounding environment. Six 3D platforms with 50 × 50, 100 × 100, and 150 × 150 μm^2^ microwells without and with the cover were used to evaluate the cytotoxicity of PTX and PEG-PTX NPs. The effect of the cover on cell proliferation was presented by the percentage of cell division, as shown in Supplementary Figures S4 and S5 and Table S1. As shown in Supplementary Figure S4, after NPC43 cells were incubated for 16 h without any drug treatment, cells could divide in all microwells without and with covers. Furthermore, the percentage of cell division was calculated as shown in Supplementary Figure S5 and Table S1, which exhibited that the cell division was inhibited in microwells with covers. However, the size of microwells did not affect cell proliferation because the percentages of cell division were similar for 50 × 50, 100 × 100, and 150 × 150 μm^2^ microwells without (~11%) and with (~7%) covers. There was no cell division after cells were treated with PTX or PEG-PTX NPs because the drugs inhibited mitosis.

**Figure 4:**
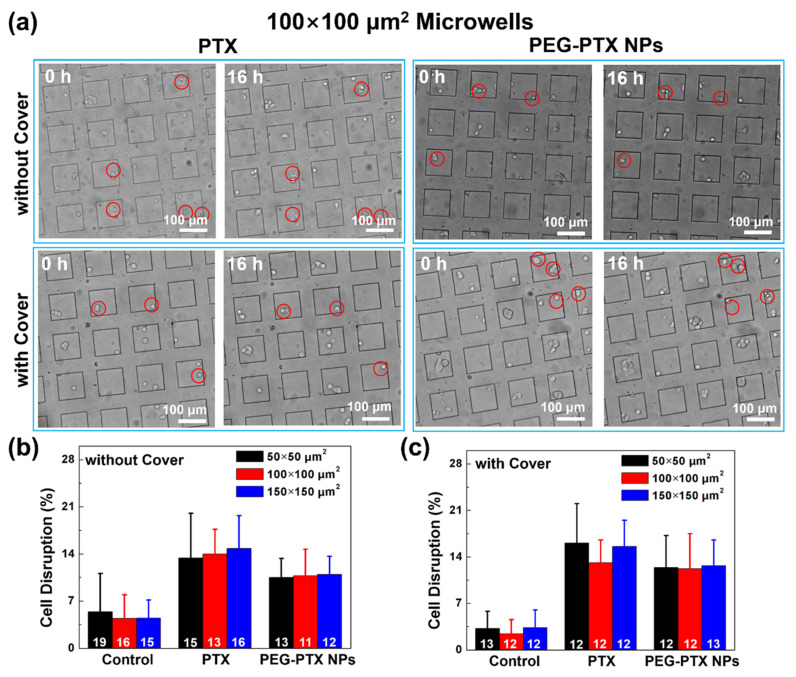
Comparison of NPC43 cell disruption ratio with different treatments. (**a**) Optical images of NPC43 cells incubated with PTX and PEG-PTX NPs for 16 h in 100 × 100 μm^2^ microwells without and with glass cover. Cell disruption highlighted in red circles. Percentage of cell disruption after PTX and PEG-PTX NP addition for 16 h in different microwells (**b**) without cover and (**c**) with cover. Number of NPC43 cells counted marked in white. Concentration of PTX and PEG-PTX NPs were both 5.85 nmol/mL.

To study the cytotoxicity of PTX and PEG-PTX NPs, the percentage of cell disruption was calculated as shown in Supplementary Table S2. Some cells ruptured after treatment with PTX or PEG-PTX NPs for 16 h in microwells of different sizes, as shown in Figure 4a and Supplementary Figures S6 and S7. As shown in Figure 4b,c, the size of microwells had no effect on cell division, so 100 × 100 μm^2^ microwells were used to further explain the results. In 100 × 100 μm^2^ microwells without a cover, the percentages of cell disruption after PTX and PEG-PTX NP addition for 16 h were 14.01 ± 3.66% and 10.77 ± 3.92%, respectively, while the percentage of cell disruption was only 4.42 ± 3.50% for cells without treatment. This result showed that PTX was more effective in treating NPC43 cells than PEG-PTX NPs, which was consistent with the MTT assay results. Furthermore, in 100 × 100 μm^2^ microwells with a cover, the percentages of cell disruption after PTX and PEG-PTX NP treatment for 16 h were 13.12 ± 3.44% and 12.23 ± 5.26%, respectively. After treatment with PEG-PTX NPs for 16 h, slightly more cells ruptured in microwells with a cover than in microwells without a cover. Similar results were obtained for 50 × 50 and 150 × 150 μm^2^ microwells, although the differences were not statistically significant.

Our findings indicate that the cytotoxicity of both PTX and PEG-PTX NPs on NPC43 cells was lower in microwells than in population-based cell viability tests with 2D culture. In 2D culture, NPC43 cell viabilities with both PTX and PEG-PTX NP treatments were lower than 75% within 16 h. However, in microwells, overall cell viability derived from cell disruptions was larger than 85%. Unfortunately, a direct comparison between 2D and microwell cultures was not possible due to cells quickly detaching and moving out of the selected imaging field on flat 2D surfaces during time-lapse imaging. Nonetheless, similar trends of cancer cell resilience variation to nanomedicine treatment in 2D and 3D culture have been previously reported [40,41]. In microwells with glass covers, the NPC43 cells were less healthy due to the inhibited nutrient supply, as reflected in their reduced proliferation rate. However, in such circumstances, the cytotoxicity of both drugs towards the NPC43 cells was lower compared to an open area. Initially, it was suspected that the glass cover was blocking the supply of drugs to the isolated microwells, but the experimental results without a glass cover were similar. Thus, the reduced cytotoxicity was majorly due to the physical confinement that isolated NPC43 cells into smaller colonies. These results also align with our previous studies [25]. 

### 3.3. NPC43 Cell Behaviors with PTX and PEG-PTX NPs Treatments

#### 3.3.1. Reduced NPC43 Migration Speed over Time with Drug Treatment

Cell migration speed is a key indicator of cell behavior, and PTX can inhibit cell motility [42,43]. Therefore, cell migration speed was monitored after treating cells with PTX and PEG-PTX NPs in different microwells, with cells on a flat surface also tested for comparison. As shown in Supplementary Figure S8, the migration speed of NPC43 cells decreased slightly after 16 h without treatment. However, after incubating with PTX, cell migration speed decreased to 0.1 μm/min after 5.4 h of treatment, defined as t_0.1_. This decrease was due to the small molecule PTX acting directly on tubulin to inhibit cell movement. In contrast, cells treated with PEG-PTX NPs had a t_0.1_ of 12.4 h, indicating that PEG-PTX NPs could continuously release PTX to slow down NPC43 cell motility.

**Figure 5:**
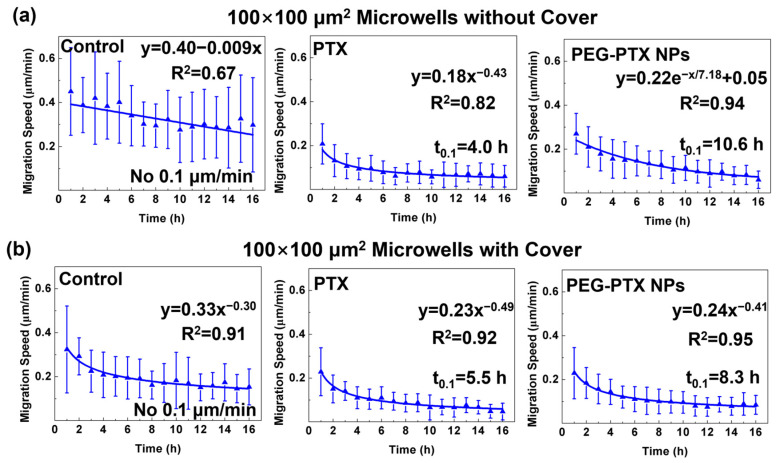
Trends of NPC43 cell migration speed in 100 × 100 μm^2^ microwells (**a**) without cover and (**b**) with cover after different treatments. These groups include control without any treatment, NPC43 cells treated with PTX, and NPC43 cells treated with PEG-PTX NPs. t_0.1_ represents time when migration speed was equal to 0.1 μm/min. Concentration of PTX and PEG-PTX NPs were both 5.85 nmol/mL.

The cell migration speed of NPC43 cells in different microwells with and without covers is shown in Figure 5 and Supplementary Figures S9 and S10. Further explanation was based on 100 × 100 μm^2^ microwells because the trends of cell migration speed were similar for different microwell sizes after the same treatment. As shown in Figure 5a, in microwells without a cover, without drug treatment, NPC43 cell migration speed decreased slightly over time as the cells gradually clustered, which agreed with the previous study [44]. Cell migration speed decreased quickly after treatment with PTX for 16 h, with a t_0.1_ of 4.0 h. On the other hand, cell migration speed decreased continuously after treatment with PEG-PTX NPs, with a t_0.1_ of 10.6 h. 

However, in 100 × 100 μm^2^ microwells with covers, the cell migration speed fell to 0.2 μm/min in the first 5 h and decreased very slowly in the following 11 h for the control group without treatment, as shown in Figure 5b. The decreased migration speed was mainly due to the lack of O_2_ and nutrient supply in the confined 3D environment [45,46]. After treatment with PTX for 16 h, the trends of migration speed in microwells with covers were similar to those in microwells without covers. However, after treatment with PEG-PTX NPs for 16 h, the trends of migration speed were different in microwells without and with covers, and two different curves were required to fit their trends. After treatment with PEG-PTX NPs, the migration speed fell rapidly in the first several hours and then maintained a very slow decline when cells were in microwells with covers. Therefore, the t_0.1_ for cells treated with PEG-PTX NPs in 100 × 100 μm^2^ microwells with covers was 8.3 h, which was 2.3 h less than t_0.1_ for cells treated with PEG-PTX NPs in 100 × 100 μm^2^ microwells without covers. These results demonstrated that covers provided additional confinement and decreased the migration speed of NPC43 cells. Cells treated with the small molecule anticancer drug PTX and PEG-PTX NPs showed different trends in cell migration speed. A similar drug-induced cell migration speed reduction was obtained in 50 × 50 μm^2^ and 150 × 150 μm^2^ microwells, as shown in Supplementary Figures S8 and S9.

#### 3.3.2. PTX Took Effect Faster than PEG-PTX NPs under Microwell Confinement

**Figure 6:**
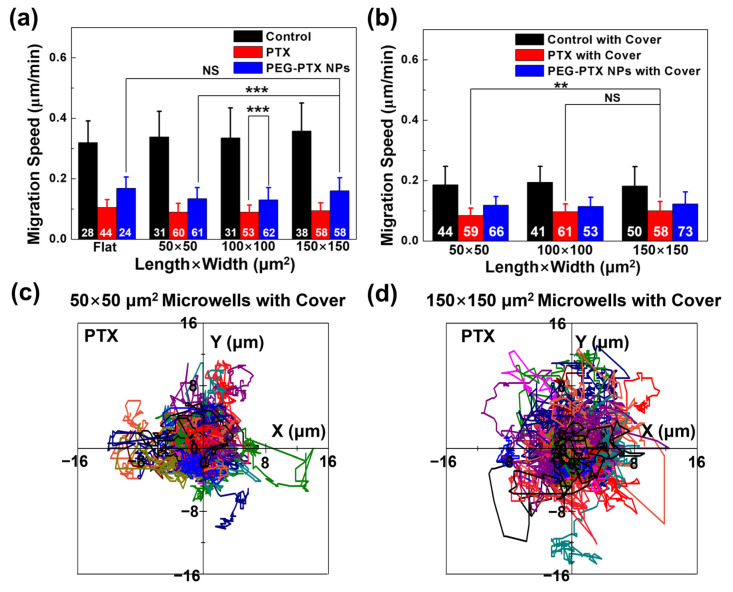
NPC43 cell migration behavior in microwells without and with cover. (**a**) Migration speed of NPC43 cells with PTX and PEG-PTX NPs treatments over 16 h in microwells (**a**) without cover and (**b**) with cover. Cell migration trajectories of NPC43 cells over 16 h with PTX added in (**c**) 50 × 50 μm^2^ microwells with cover and (**d**) 150 × 150 μm^2^ microwells with cover. Starting points of cell migration trajectories are (0, 0), trajectories of individual cells labeled with different colors. One-way ANOVA and Tukey’s post-hoc tests, NS—not significant, ** *p* < 0.01, and *** *p* < 0.001. Number of NPC43 cells counted in white. Concentration of PTX and PEG-PTX NPs were both 5.85 nmol/mL.

The average migration speed of NPC43 cells on a flat surface and in different microwells is shown in Supplementary Table S3. As shown in Figure 6a, the average migration speeds of cells without any treatment on the flat surface and in different microwells without covers were similar, with speeds over 0.3 μm/min. After treatment with PTX or PEG-PTX NPs for 16 h, the average migration speed significantly decreased. The average migration speed was only about 0.1 μm/min after treatment with PTX, while the average migration speed of cells treated with PEG-PTX NPs was faster, indicating that PTX was more effective than PEG-PTX NPs in treating NPC43 cells in 16 h. After treatment with PEG-PTX NPs in 150 × 150 μm^2^ microwells for 16 h, the average migration speed was 0.16 ± 0.04 μm/min. However, the average migration speed was 0.13 ± 0.04 μm/min for cells in 50 × 50 μm^2^ microwells with the same treatment, indicating that NPC43 cells treated with PEG-PTX NPs had a faster speed in larger microwells. The average migration speed was also calculated in microwells with covers, as shown in Figure 6b. The cover decreased the migration speed because it inhibited the exchange of nutrients, making the cells unhealthy. For control groups, the average migration speeds were about 0.20 μm/min in all microwells with covers, which were slower than those in microwells without covers with speeds over 0.3 μm/min. In microwells with covers, NPC43 cells treated with PTX still had the slowest speed among other groups, including control without any treatment and NPC43 cells treated with PEG-PTX NPs. However, for the NPC43 cells treated with PTX, there was no difference in the average migration speed in microwells without and with covers, as shown in Supplementary Figure S11a. There was no statistically significant difference observed in cell migration speed when treated with PEG-PTX NPs in glass-covered microwells of different sizes. The additional confinement by the cover may have limited the supply of PTX into the microwells, slightly decreasing the cytotoxicity, but PTX was still toxic enough to decrease the migration speed. It was a balance of PTX toxicity and the effect of 3D confinement. After treatment with PTX, the t_0.1_ for microwells with covers was larger than t_0.1_ for microwells without covers, as shown in Figure 5 and Supplementary Figures S9 and S10. For the NPC43 cells treated with PEG-PTX NPs in microwells with covers, the average speed was lower than those in microwells without covers, especially in 150 × 150 μm^2^ microwells, as shown in Supplementary Figure S11b. In 150 × 150 μm^2^ microwells without covers, PEG-PTX NPs needed a longer time to release PTX and inhibit cell movement, with t_0.1_ of 12.9 h, as shown in Supplementary Figure S9a. Additionally, the cover decreased the migration speed in the first 5 h, so the decrease in migration speed was a combination effect of nanomedicine and confinement. The trajectories of NPC43 cells without any drug treatment and treated with PTX in 50 × 50 and 150 × 150 μm^2^ microwells are shown in Supplementary Figure S12 and Figure 6c,d, respectively. As shown in Supplementary Figure S12, in microwells with covers, the moving range of NPC43 cells without any drug treatment was smaller than that in microwells without covers, but there was no difference in the moving range of NPC43 cells without any drug treatment in microwells with different sizes. After treatment with PTX, the moving range of NPC43 cells was reduced, and the moving range of cells in 150 × 150 μm^2^ microwells was slightly bigger than that in 50 × 50 μm^2^ microwells, as shown in Figure 6b,c. Cells without any treatment were healthy and possessed normal speed to move and change directions. In complete confinement with covers on top of microwells, the speed of cells decreased, and cells could not move as far as cells in microwells without covers. After treatment with PTX, cells were too unhealthy to move or change directions. In smaller microwells, it was easier for NPC43 cells treated with PTX to touch the sidewalls of the microwells, and the cells could not move further, leading to a smaller moving range.

#### 3.3.3. PEG-PTX NPs Had More Stable Performance than PTX under Isolation and Confinement

**Figure 7:**
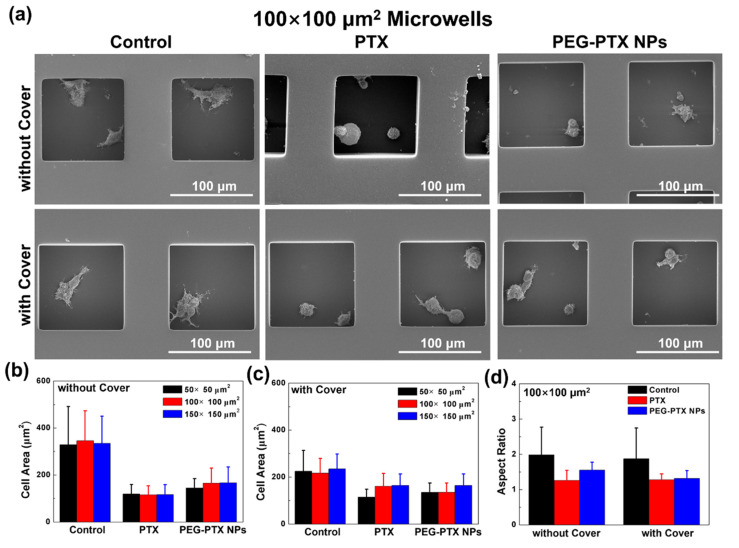
NPC43 cell morphology after various treatments. (**a**) Scanning electron micrographs of NPC43 cells with PTX and PEG-PTX NPs added over 16 h in 100 × 100 μm^2^ microwells without and with cover. Cell area after cells treated with PTX and PEG-PTX NPs for 16 h in microwells (**b**) without cover and (**c**) with cover. (**d**) Cell aspect ratio after NPC43 cells treated with PTX and PEG-PTX NPs for 16 h in 100 × 100 μm^2^ microwells without and with cover. Number of NPC43 cells counted in white. Concentration of PTX and PEG-PTX NPs were both 5.85 nmol/mL.

The morphology of NPC43 cells in microwells was observed using SEM. As shown in Figure 7 and Supplementary Figures S13 and S14, NPC43 cells without any treatment in microwells without and with covers were elongated and had many extended filopodia. However, after treatment with PTX for 16 h, NPC43 cells became rounded and had few filopodia. The NPC43 cells treated with PEG-PTX NPs were elliptical with a few filopodia. To analyze the change in cell morphology, the cell area and aspect ratio were evaluated, as shown in Supplementary Tables S4 and S5. As shown in Figure 7b, NPC43 cells without any treatment had the largest cell area, while NPC43 cells treated with PTX had the smallest cell area. The cell area for NPC43 cells treated with PEG-PTX NPs was slightly larger than that for NPC43 cells treated with PTX. In microwells with covers, the cell area was about 200 μm^2^, which was smaller than the cell area of over 300 μm^2^ for NPC43 cells in microwells without covers, as shown in Figure 7b,c. The cell area was similar for the NPC43 cells treated with PTX and PEG-PTX NPs. In 100 × 100 μm^2^ microwells, the cell area was 345 ± 127 μm^2^ for control without covers, 115 ± 39 μm^2^ for PTX without covers, 165 ± 64 μm^2^ for PEG-PTX NPs without covers, 216 ± 63 μm^2^ for control with covers, 139 ± 44 μm^2^ for PTX with covers, and 135 ± 44 μm^2^ for PEG-PTX NPs with covers. When the microwells were concealed by glass covers, the reduced supply of O_2_ and nutrients over time resulted in fewer healthy cells with reduced cell area in the control groups, as shown in Figure 7a,b. However, the glass cover also hindered the supply of drugs from fresh medium. The effect of PTX quickly wore off with the glass cover blocking the supply, and the cells were more spread out compared to the scenario without a glass cover. On the other hand, PEG-PTX NPs took effect more slowly but had a higher interaction efficacy, and the results were not significantly hindered by the limited drug supply due to glass cover concealment. Furthermore, the aspect ratio of cells, which illustrated cell elongation, was calculated. As shown in Figure 7d, in 100 × 100 μm^2^ microwells, the aspect ratios were 1.98 ± 0.78 for control without covers, 1.26 ± 0.28 for PTX without covers, 1.55 ± 0.22 for PEG-PTX NPs without covers, 1.87 ± 0.87 for control with covers, 1.27 ± 0.17 for PTX with covers, and 1.36 ± 0.23 for PEG-PTX NPs with covers. These results were consistent with the average speed dependence on the microwell size and the presence of the cover. In the control group without covers, NPC43 cells moved normally and elongated with the highest aspect ratio. After treatment with PTX, NPC43 cells became rounded, and the aspect ratio was the lowest. Supplementary Figure S15 shows that similar results were obtained in 50 × 50 and 150 × 150 μm^2^ microwells, independent of the size of the microwells. 

In microwells without covers, PTX had better overall performance than PEG-PTX NPs, thanks to the abundant drug supply through medium exchange. However, when the microwells were confined by a glass cover to limit the supply of fresh medium with drug, PTX quickly depleted, leading to an obvious decline in performance. Conversely, PEG-PTX-NPs had much more stable drug release, and the drug effect appeared unaffected by the obstructed drug supply. Previous studies have shown that 3D-cultured cancer spheroids are more resilient to both PTX [47] and NP [48,49] treatments. However, the presence of NPs could enhance drug efficacy towards 3D cancer tissues [49]. The glass-covered microwells in this study provided a similar environment to the 3D cancer spheroid, where the drug supply was limited by physical barriers. Compared to spheroid culture, the unique advantage of using concealed microwells is that cells can be seeded with low density, allowing for more precise monitoring of single-cell response to drug treatment that can be continuously and conveniently monitored. 

## 4. Conclusions

In this study, we fabricated 3D microwells with and without covers to investigate the interplay between the cytotoxicity of PEG-PTX NPs and cell behaviors. Initially, we successfully synthesized stable PEG-PTX NPs with a diameter of approximately 110 nm to treat NPC43 cells. Our results showed that at short times, the small molecule drug PTX was more effective in treating NPC43 cells than PEG-PTX NPs. However, after 64 h of treatment, the effects were similar according to the MTT assays. Within 16 h, NPC43 cell viability was higher when treated with either PTX or PEG-PTX NPs in microwells than in 2D culture. This suggests that the extracellular matrix in NPC tumors may hinder drug performance through a similar confinement effect. To evaluate the cytotoxicity of PTX and PEG-PTX NPs, we compared their effects on cell disruption, cell motility, and cell morphology in microwells with and without glass cover concealments. Results indicate that PTX was more cytotoxic than PEG-PTX NPs without concealments in the first 16 h, consistent with the expected faster action of small molecule drugs. However, when microwells were concealed, cancer cells experienced a similar scenario to a 3D spheroid culture, with restricted O_2_ and nutrient exchange and drug delivery due to physical barriers. As a result, the cytotoxicity of PTX was largely hindered, while the performance of PEG-PTX NPs was more stable regardless of the limited drug supply. Overall, our study presents a novel approach to evaluating the relationship between nanomedicine, 3D confinement, and cell behaviors of isolated small cancer cell colonies in vitro. In the future, the inclusion of mixed cultures of cancer cells and normal cells from surrounding tissues could provide a better evaluation of cancer-associated heterogeneous cell interactions and potential side effects after drug release. While this approach may not fully replicate the in vivo scenario, as well as 3D cultured cancer spheroid models for drug testing, it offers a simpler and more convenient method for in vitro evaluation that focuses on single-cell analysis. This approach provides valuable insights for the development and testing of new nanomedicines.

## Data Availability

Data contained within the article or Supplementary Materials. Original data available on request from corresponding author.

## Supplementary Materials

The following supporting information can be downloaded at: https://www.mdpi.com/article/10.3390/jfb14060322/s1, Table S1: Percentage of cell disruption for cells without any treatment for 16 h in different microwells; Table S2: Percentage of cell disruption after PTX and PEG-PTX NP addition for 16 h in different microwells; Table S3: Migration speed of NPC43 cells with PTX and PEG-PTX NP treatments over 16 h in different microwells and on flat surface; Table S4: Cell area after cells treated with PTX and PEG-PTX NPs for 16 h in different microwells; Table S5: Cell aspect ratio after NPC43 cells treated with PTX and PEG-PTX NPs for 16 h in different microwells; Figure S1: Size distribution and stability of PEG-PTX NPs in cell culture medium measured by dynamic light scattering (DLS); Figure S2: Confocal laser scanning micrographs of NPC43 cells incubated with PTX and PEG-PTX NPs for 16 h in 50 × 50 μm^2^ microwells; Figure S3: Confocal laser scanning micrographs of NPC43 cells incubated with PTX and PEG-PTX NPs for 16 h in 150 × 150 μm^2^ microwells; Figure S4: Micrographs of NPC43 cells without any treatment for 16 h in microwells without and with cover. Figure S5: Percentage of cell disruption for cells without any treatment for 16 h in different microwells; Figure S6: Micrographs of NPC43 cells incubated with PTX or PEG-PTX NPs for 16 h in 50 × 50 μm^2^ microwells; Figure S7: Micrographs of NPC43 cells incubated with PTX or PEG-PTX NPs for 16 h in 150 × 150 μm^2^ microwells; Figure S8: Trends of NPC43 cell migration speed on flat surface after different treatments; Figure S9: Trends of NPC43 cell migration speed in 50 × 50 μm^2^ microwells; Figure S10: Trends of NPC43 cell migration speed in 150 × 150 μm^2^ microwells; Figure S11: NPC43 cell migration behavior in microwells with and without cover; Figure S12: Cell migration trajectories of NPC43 cells over 16 h without any treatment in 50 × 50 and 150 × 150 μm^2^ microwells without and with cover; Figure S13: Scanning electron micrographs of NPC43 cells with PTX and PEG-PTX NPs added over 16 h in 50 × 50 μm^2^ microwells without and with cover; Figure S14: Scanning electron micrographs of NPC43 cells with PTX and PEG-PTX NPs added over 16 h in 150 × 150 μm^2^ microwells without and with cover; Figure S15: Cell aspect ratio after NPC43 cells treated with PTX and PEG-PTX NPs for 16 h.
