# Supplementary material for: PEGylated Paclitaxel Nanomedicine Meets 3D Confinement: Cytotoxicity and Cell Behaviors
Source: J Funct Biomater. 2023 Jun 15;14(6):322. doi: 10.3390/jfb14060322 (PMC10298934; doi:10.3390/jfb14060322)
Supplement: Supplementary file 1 [file jfb-14-00322-s001.zip › jfb-2435337-supplementary.pdf]

Supporting Information

# PEGylated Paclitaxel Nanomedicine Meets 3D Confinement: Cytotoxicity and Cell Behaviors

*Wenhai Lin,[1,2,†] Yuanhao Xu,[1,2,†] Xiao Hong,[1,2] and Stella W. Pang [1,2,*]*

1 Department of Electrical Engineering, City University of Hong Kong, Hong Kong, China

2 Centre for Biosystems, Neuroscience, and Nanotechnology, City University of Hong Kong, Hong Kong, China

* Correspondence: pang@cityu.edu.hk

† These authors contributed equally to this work.

**Supplementary Table S1.** Percentage of cell disruption for cells without any treatment for 16 h in different microwells.

| Microwell (μm$^2$) | without cover (%) | with cover (%) |
|---|---|---|
| 50×50 | 11.63±5.79 | 7.26±2.62 |
| 100×100 | 11.43±4.57 | 6.61±2.65 |
| 150×150 | 10.47±3.65 | 7.65±2.19 |

**Supplementary Table S2**. Percentage of cell disruption after PTX and PEG-PTX NPs addition for 16 h in different microwells. Concentration of PTX and PEG-PTX NPs were both 5.85 nmol/mL.

| Microwell (μm$^2$) | | Control (%) | PTX (%) | PEG-PTX NPs (%) |
|---|---|---|---|---|
| without cover | 50×50 | 5.69±5.43 | 13.40±6.62 | 10.50±2.84 |
| | 100×100 | 4.42±3.50 | 14.01±3.66 | 10.77±3.92 |
| | 150×150 | 4.50±2.67 | 14.82±4.86 | 10.96±2.68 |
| with cover | 50×50 | 3.21±2.58 | 16.08±5.94 | 12.41±4.80 |
| | 100×100 | 2.41±2.15 | 13.12±3.44 | 12.23±5.26 |
| | 150×150 | 3.37±2.63 | 15.56±3.96 | 12.68±3.87 |

**Supplementary Table S3**. Migration speed of NPC43 cells with PTX and PEG-PTX NPs treatments over 16 h in different microwells and on flat surface. Concentration of PTX and PEG-PTX NPs were both 5.85 nmol/mL.

| Microwell (μm$^2$) | | Control (μm/min) | PTX (μm/min) | PEG-PTX NPs (μm/min) |
|---|---|---|---|---|
| without cover | Flat surface | 0.32±0.07 | 0.10±0.03 | 0.17±0.04 |
| | 50×50 | 0.34±0.08 | 0.09±0.03 | 0.13±0.04 |
| | 100×100 | 0.33±0.06 | 0.09±0.02 | 0.13±0.04 |
| | 150×150 | 0.36±0.09 | 0.09±0.03 | 0.16±0.04 |
| with cover | 50×50 | 0.32±0.07 | 0.08±0.02 | 0.12±0.03 |
| | 100×100 | 0.32±0.07 | 0.10±0.03 | 0.11±0.03 |
| | 150×150 | 0.32±0.07 | 0.10±0.03 | 0.12±0.04 |

**Supplementary Table S4**. Cell area after cells treated with PTX and PEG-PTX NPs for 16 h in different microwells. Concentration of PTX and PEG-PTX NPs were both 5.85 nmol/mL.

| Microwell (μm$^2$) | | Control (μm$^2$) | PTX (μm$^2$) | PEG-PTX NPs (μm$^2$) |
|---|---|---|---|---|
| without cover | 50×50 | 329±162 | 119±41 | 145±40 |
| | 100×100 | 345±127 | 115±39 | 165±64 |
| | 150×150 | 334±115 | 116±43 | 166±68 |
| with cover | 50×50 | 225±88 | 114±34 | 135±39 |
| | 100×100 | 216±63 | 139±44 | 135±44 |
| | 150×150 | 234±64 | 152±33 | 140±52 |

**Supplementary Table S5**. Cell aspect ratio after NPC43 cells treated with PTX and PEG-PTX NPs for 16 h in different microwells. Concentration of PTX and PEG-PTX NPs were both 5.85 nmol/mL.

| Microwell ($\mu m^2$) | | Control | PTX | PEG-PTX NPs |
|---|---|---|---|---|
| without cover | 50×50 | 1.77±0.65 | 1.31±0.20 | 1.55±0.35 |
| | 100×100 | 1.98±0.78 | 1.26±0.28 | 1.55±0.22 |
| | 150×150 | 1.79±0.58 | 1.22±43 | 1.60±0.30 |
| with cover | 50×50 | 1.93±0.78 | 1.22±0.18 | 1.31±0.22 |
| | 100×100 | 1.87±0.87 | 1.27±0.17 | 1.36±0.23 |
| | 150×150 | 1.71±0.47 | 1.21±0.16 | 1.29±0.20 |

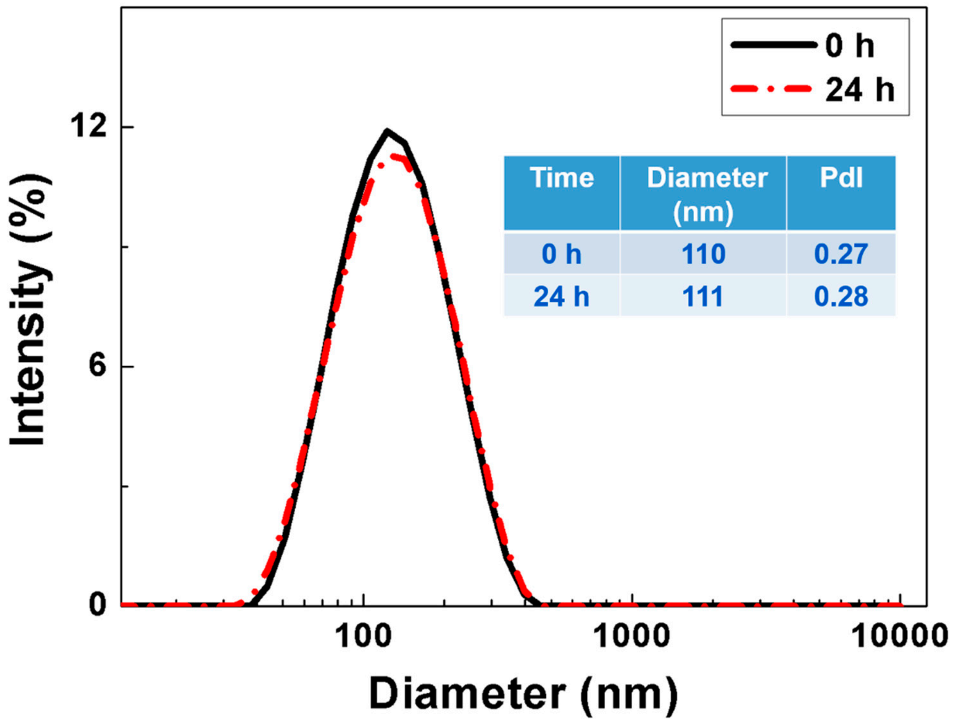


**Supplementary Figure S1**. Size distribution and stability of PEG-PTX NPs in cell culture medium measured by dynamic light scattering (DLS).

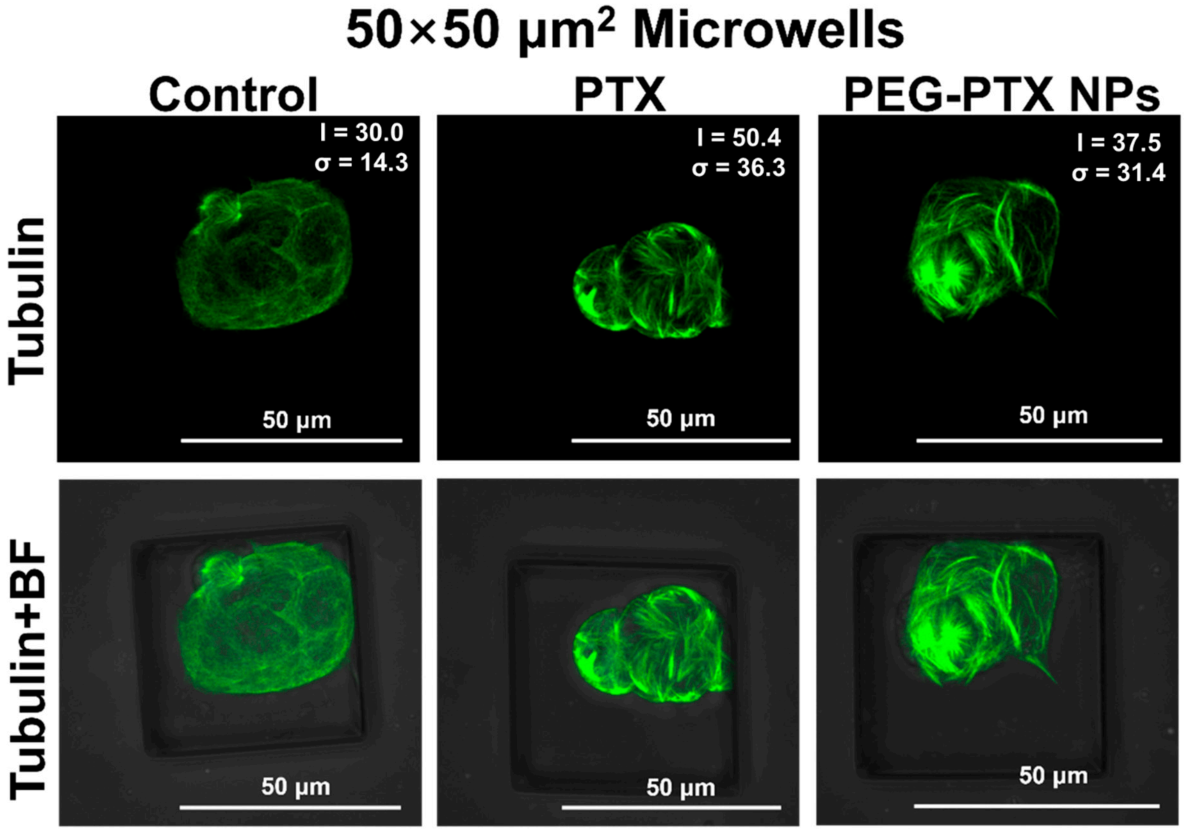


**Supplementary Figure S2**. Confocal laser scanning micrographs of NPC43 cells incubated with PTX and PEG-PTX NPs for 16 h in 50×50 $\mu m^2$ microwells. Tubulin in cells was stained by tubulin-tracker green (green fluorescence), and overlays of tubulin and bright field (BF) images. Average value (I) and standard deviation (σ) of fluorescent intensity were larger after drug treatment. Concentration of PTX and PEG-PTX NPs were both 5.85 nmol/mL.

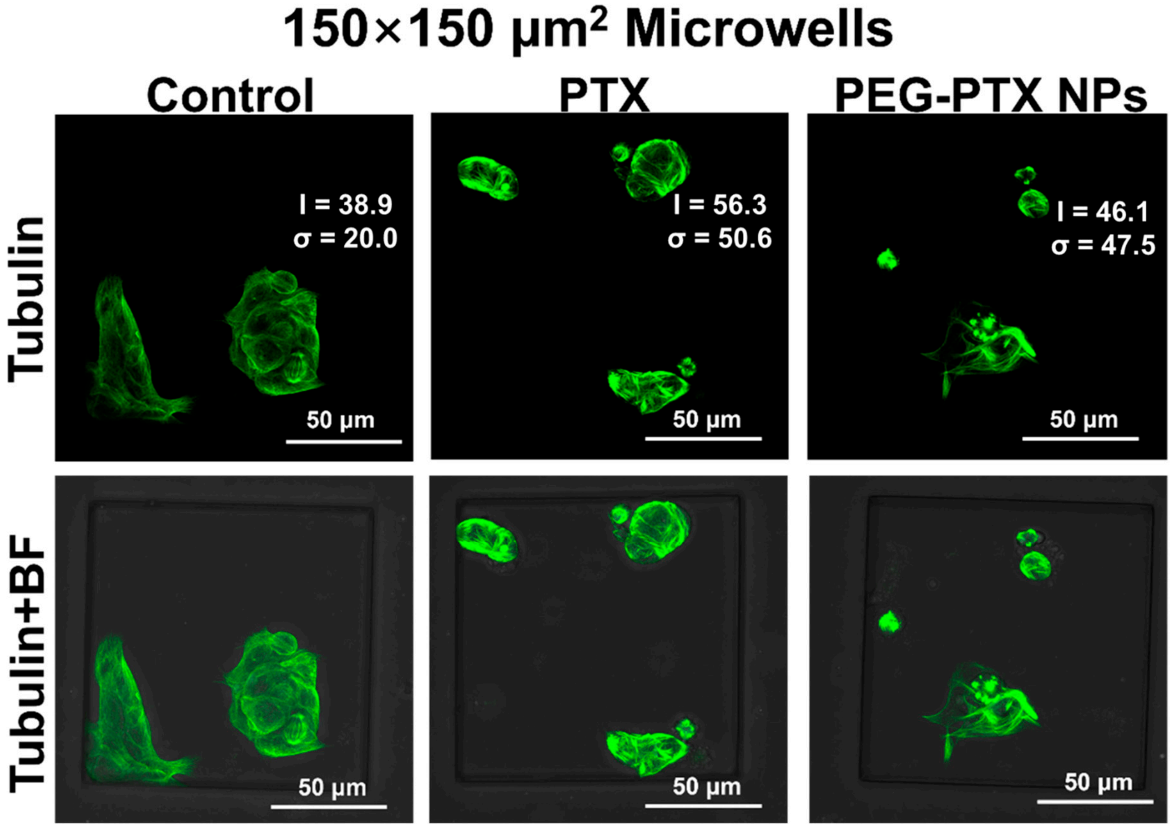


**Supplementary Figure S3**. Confocal laser scanning micrographs of NPC43 cells incubated with PTX and PEG-PTX NPs for 16 h in 150×150 μm$^2$ microwells. Tubulin in cells was stained by tubulin-tracker green (green fluorescence), and overlays of Tubulin and BF images. Average value (I) and standard deviation (σ) of fluorescent intensity were larger after drug treatment. Concentration of PTX and PEG-PTX NPs were both 5.85 nmol/mL.

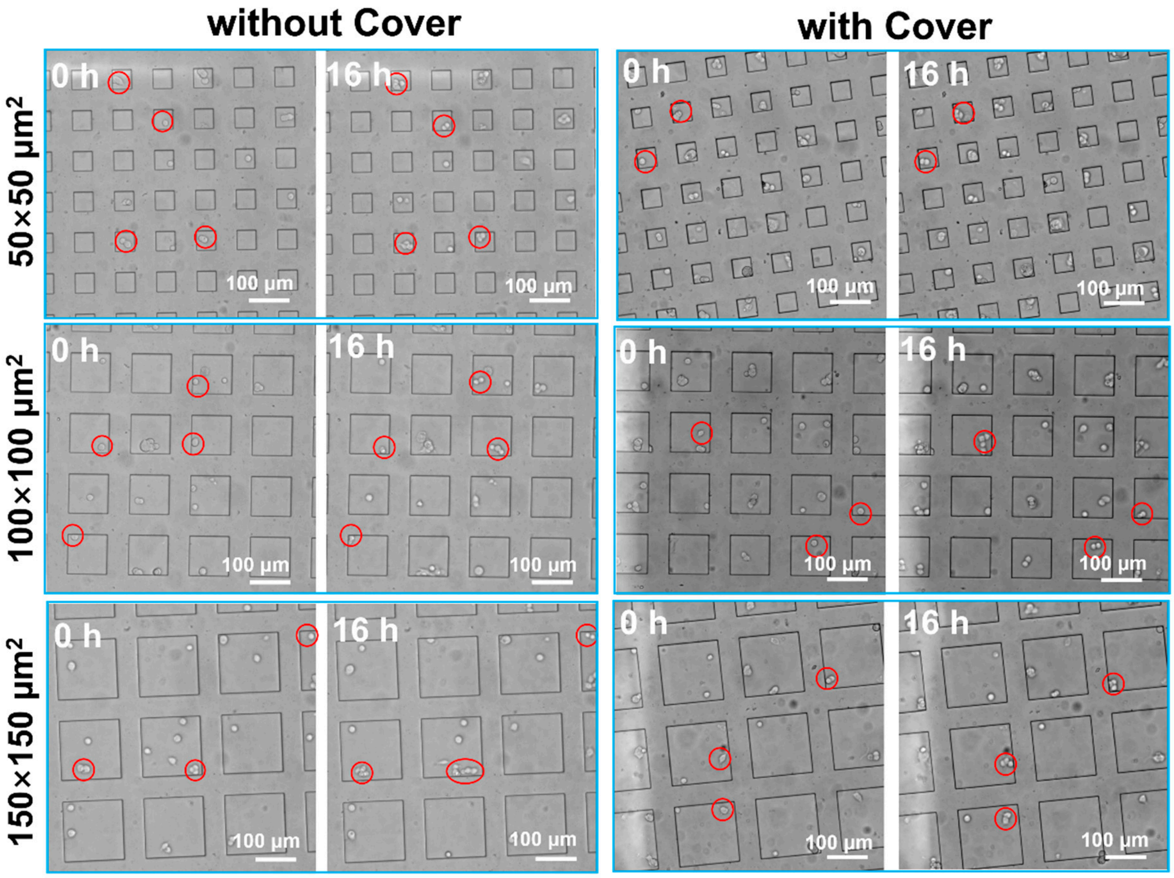


**Supplementary Figure S4**. Micrographs of NPC43 cells without any treatment for 16 h in microwells without and with cover. Cell division was highlighted in red circles.

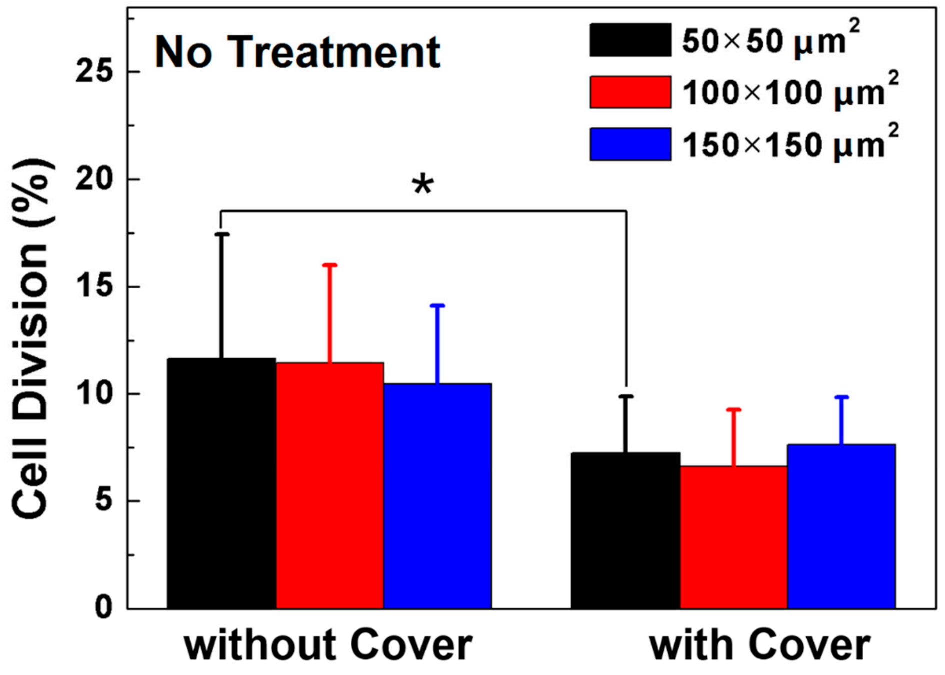


**Supplementary Figure S5**. Percentage of cell disruption for cells without any treatment for 16 h in different microwells. One-way ANOVA and Tukey's post hoc tests, *p <0.05. Concentration of PTX and PEG-PTX NPs were both 5.85 nmol/mL.

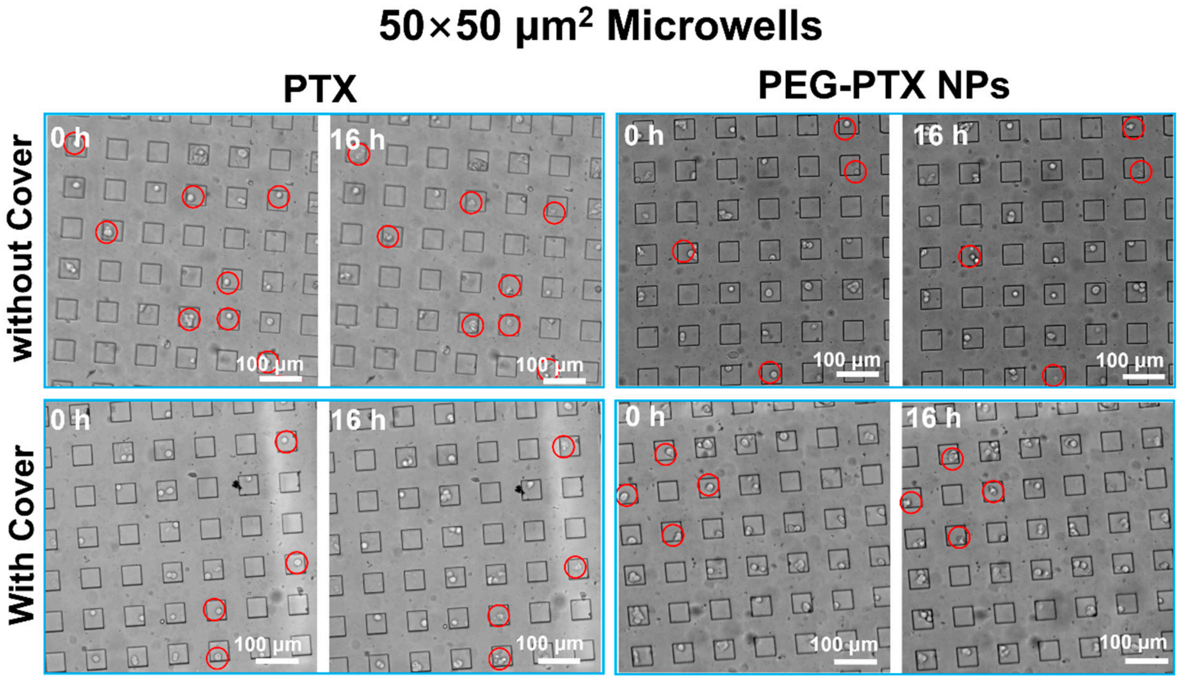


**Supplementary Figure S6**. Micrographs of NPC43 cells incubated with PTX or PEG-PTX NPs for 16 h in 50×50 μm$^2$ microwells. Cell disruption was highlighted in red circles. Concentration of PTX and PEG-PTX NPs were both 5.85 nmol/mL.

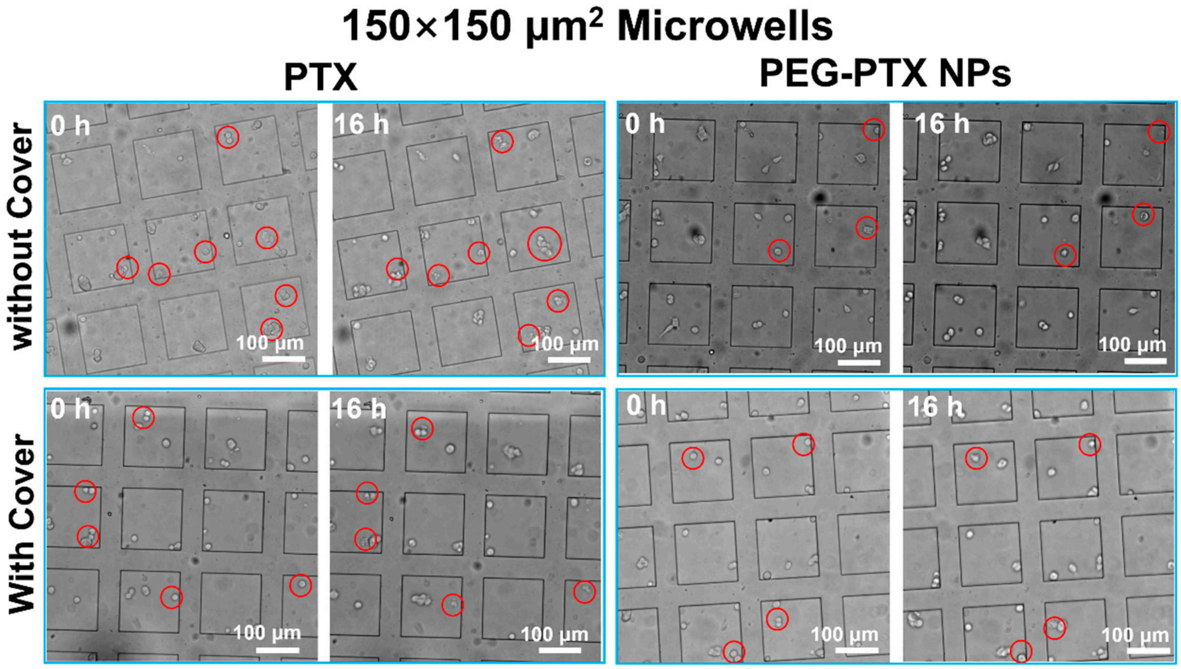


**Supplementary Figure S7**. Micrographs of NPC43 cells incubated with PTX or PEG-PTX NPs for 16 h in 150×150 μm$^2$ microwells. Cell disruption was highlighted in red circles. Concentration of PTX and PEG-PTX NPs were both 5.85 nmol/mL.

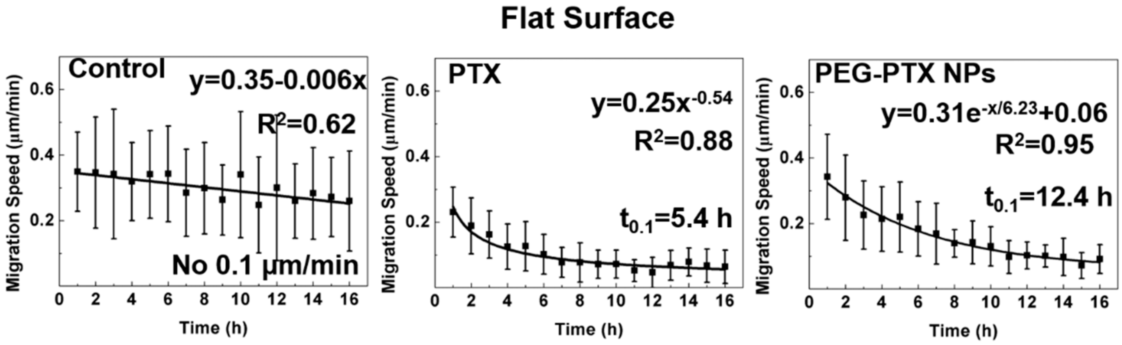


**Supplementary Figure S8**. Trends of NPC43 cell migration speed on flat surface after different treatments. These groups include control without any treatment, NPC43 cells treated with PTX, and NPC43 cells treated with PEG-PTX NPs. $t_{0.1}$ represents time when migration speed was equal to 0.1 μm/min. Concentration of PTX and PEG-PTX NPs were both 5.85 nmol/mL.

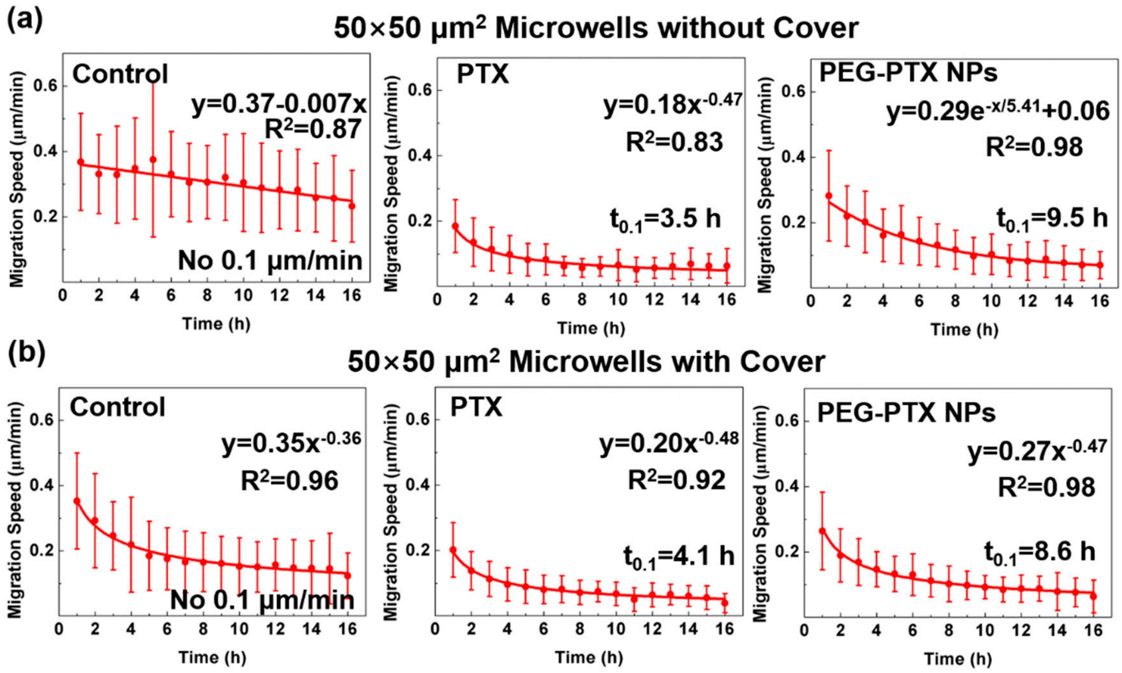


**Supplementary Figure S9**. Trends of NPC43 cell migration speed in 50×50 μm$^2$ microwells (a) without cover and (b) with cover after different treatments. These groups include control without any treatment, NPC43 cells treated with PTX, and NPC43 cells treated with PEG-PTX NPs. $t_{0.1}$ represents time when migration speed was equal to 0.1 μm/min. Concentration of PTX and PEG-PTX NPs were both 5.85 nmol/mL.

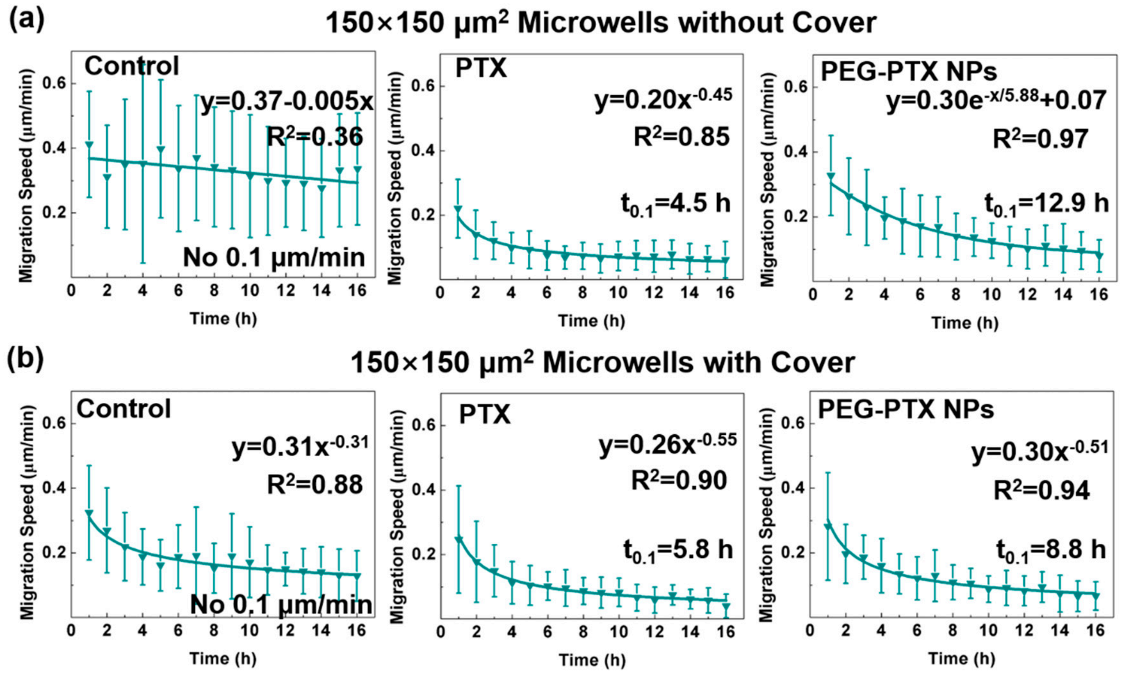


**Supplementary Figure S10**. Trends of NPC43 cell migration speed in 150×150 μm$^2$ microwells (a) without cover and (b) with cover after different treatments. These groups include control without any treatment, NPC43 cells treated with PTX, and NPC43 cells treated with PEG-PTX NPs. $t_{0.1}$ represents time when migration speed was equal to 0.1 μm/min. Concentration of PTX and PEG-PTX NPs were both 5.85 nmol/mL.

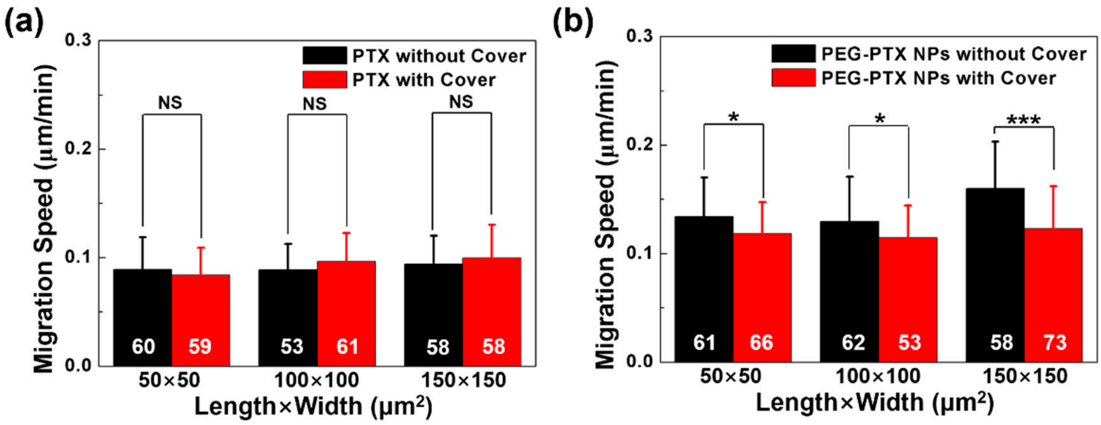


**Supplementary Figure S11**. NPC43 cell migration behavior in microwells with and without cover. (a) Migration speed of NPC43 cells with (a) PTX and (b) PEG-PTX NPs treatments over 16 h in microwells without cover and with cover. One-way ANOVA and Tukey's post hoc test, NS – not significant, *p <0.05, and ***p <0.001. Number of NPC43 cells counted is marked in white. Concentration of PTX and PEG-PTX NPs were both 5.85 nmol/mL.

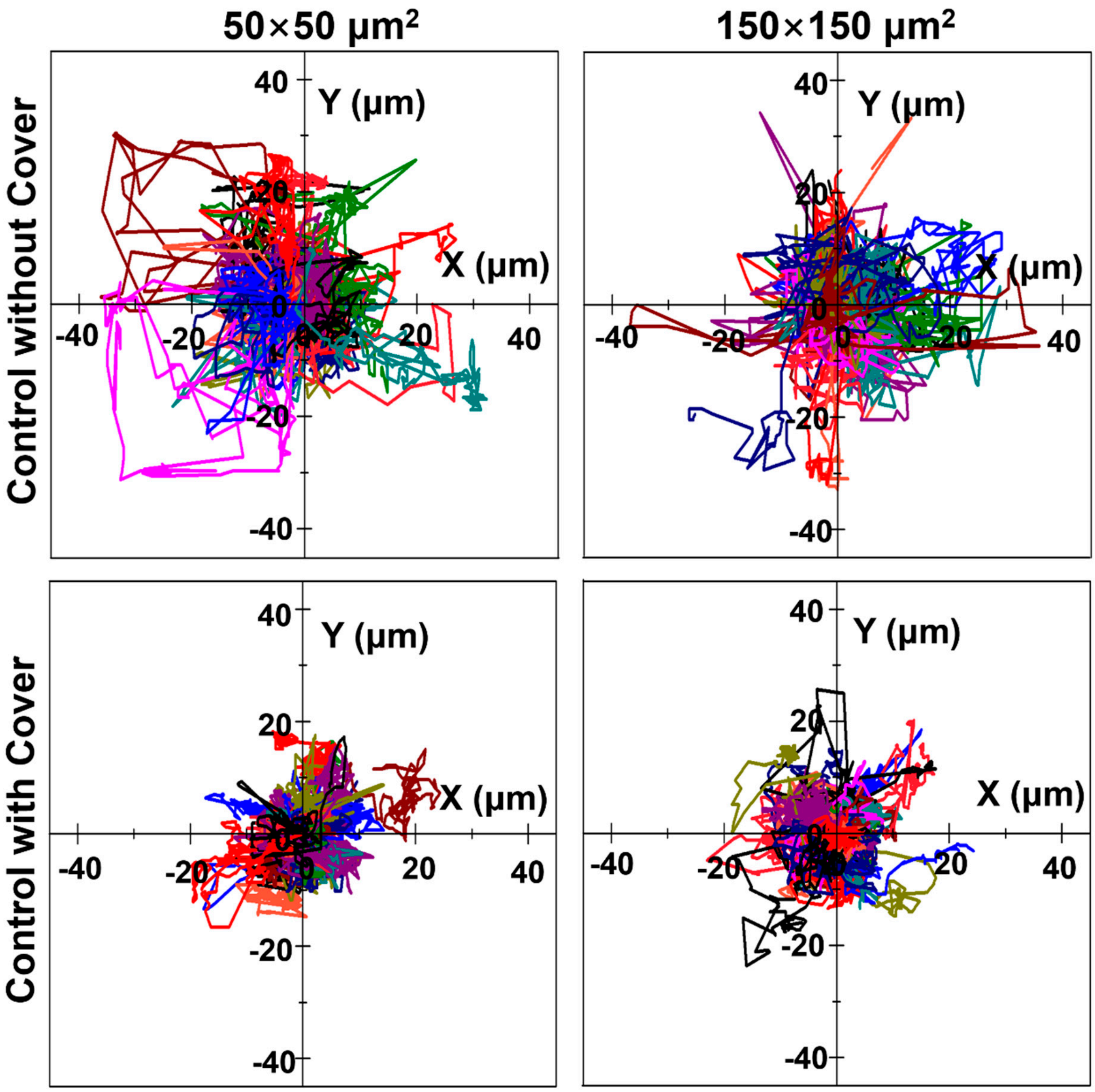


**Supplementary Figure S12**. Cell migration trajectories of NPC43 cells over 16 h without any treatment in 50×50 and 150×150 μm$^2$ microwells without and with cover. Starting points of cell migration trajectories are (0, 0).

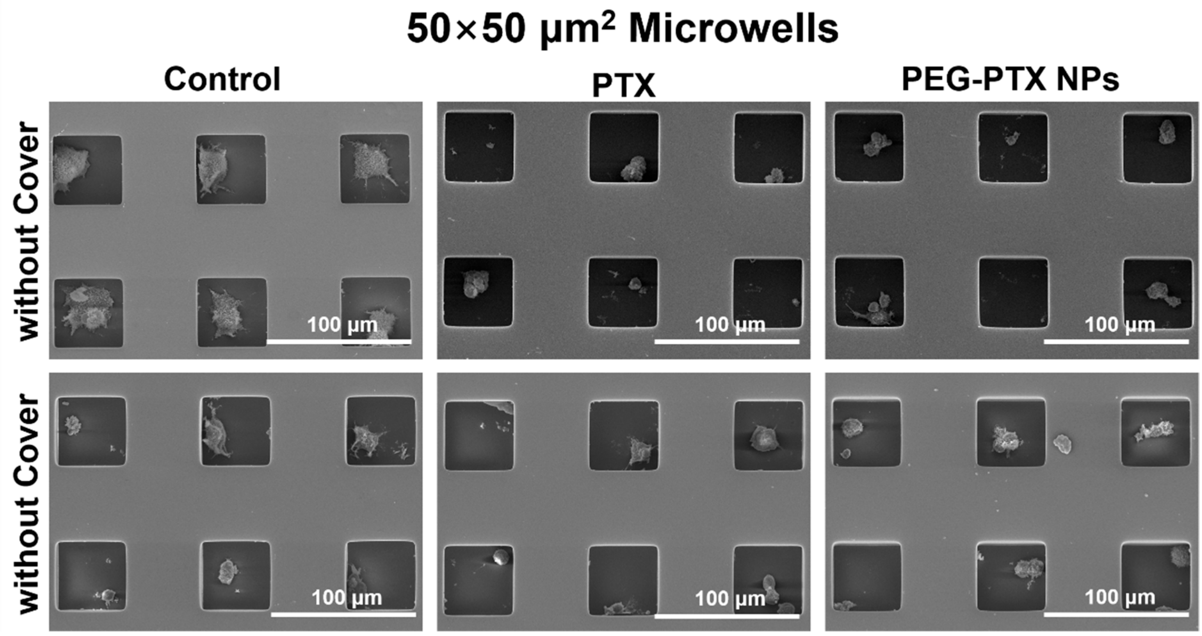


**Supplementary Figure S13**. Scanning electron micrographs of NPC43 cells with PTX and PEG-PTX NPs added over 16 h in 50×50 μm$^2$ microwells without and with cover. Concentration of PTX and PEG-PTX NPs were both 5.85 nmol/mL.

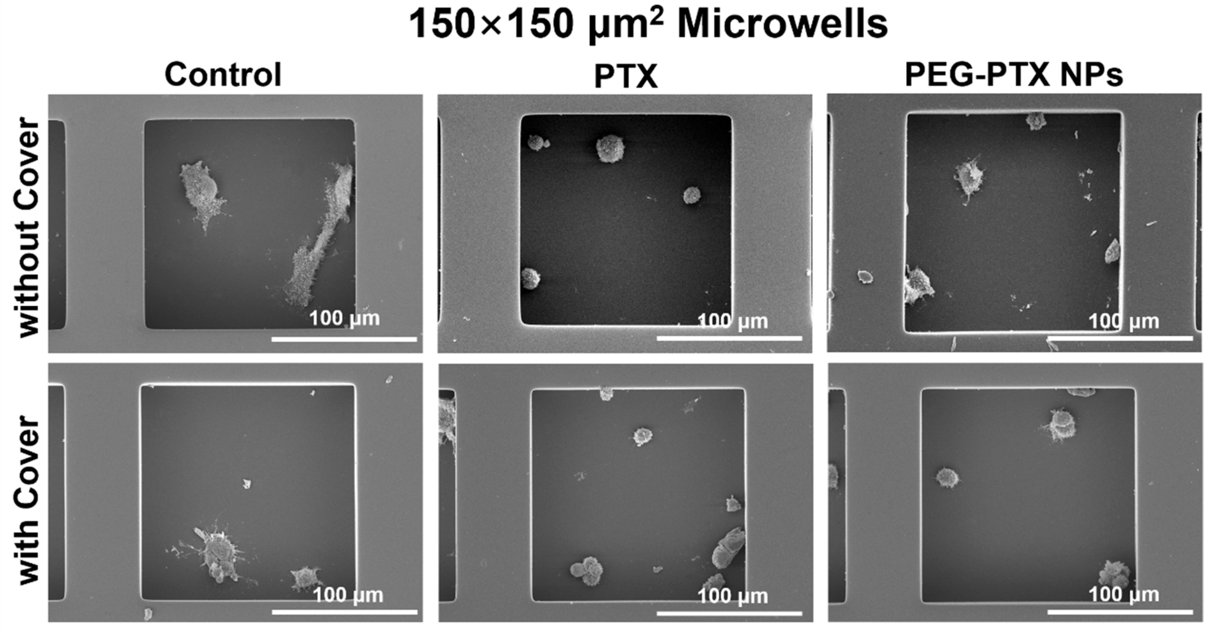


**Supplementary Figure S14**. Scanning electron micrographs of NPC43 cells with PTX and PEG-PTX NPs added over 16 h in 150×150 μm$^2$ microwells without and with cover. Concentration of PTX and PEG-PTX NPs were both 5.85 nmol/mL.

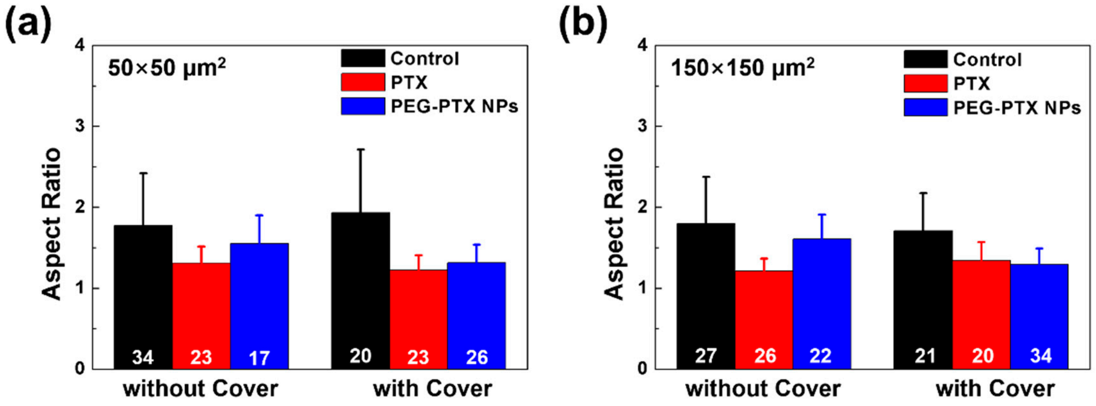


**Supplementary Figure S15**. Cell aspect ratio after NPC43 cells treated with PTX and PEG-PTX NPs for 16 h in (a) 50×50 μm$^2$ and (b) 150×150 μm$^2$ microwells without and with cover. Number of NPC43 cells counted is marked in white. Concentration of PTX and PEG-PTX NPs were both 5.85 nmol/mL.
